# Determinants of province-based health service utilization according to Andersen’ s Behavioral Model: a population-based spatial panel modeling study

**DOI:** 10.1186/s12889-023-15885-4

**Published:** 2023-05-27

**Authors:** Yu Xin, Xiaohui Ren

**Affiliations:** 1 Department of Science and Technology, West China Hospital, Sichuan University, Chengdu, Sichuan China; 2 West China School of Public Health and West China Fourth Hospital, Sichuan University, Chengdu, Sichuan China

**Keywords:** Health Service Utilization, China, Andersen’ s Behavioral Model, Spatial spillover effects, Geography

## Abstract

**Objective:**

The Andersen’ s Behavioral Model was used to explore the impact of various factors on the utilization of health services. The purpose of this study is to establish a provincial-level proxy framework for the utilization of health services from a spatial perspective, based on the influencing factors of the Andersen’ s Behavioral Model.

**Method:**

Provincial-level health service utilization was estimated by the annual hospitalization rate of residents and the average number of outpatient visits per year from China Statistical Yearbook 2010–2021. Exploring the relevant influencing factors of health service utilization using the spatial panel Durbin model. Spatial spillover effects were introduced to interpret the direct and indirect effects influenced by the proxy framework for predisposing, enabling, and need factors on health services utilization.

**Results:**

From 2010 − 2020, the resident hospitalization rate increased from 6.39% ± 1.23% to 15.57% ± 2.61%, and the average number of outpatient visits per year increased from 1.53 ± 0.86 to 5.30 ± 1.54 in China. For different provinces, the utilization of health services is uneven. The results of the Durbin model show that locally influencing factors were statistically significantly related to an increase in the resident hospitalization rate, including the proportion of 65-year-olds, GDP per capita, percentage of medical insurance participants, and health resources index, while statistically related to the average number of outpatient visits per year, including the illiteracy rate and GDP per capita. Direct and indirect effects decomposition of resident hospitalization rate associated influencing factors demonstrated that proportion of 65-year-olds, GDP per capita, percentage of medical insurance participants, and health resources index not only affected local resident hospitalization rate but also exerted spatial spillover effects toward geographical neighbors. The illiteracy rate and GDP per capita have significant local and neighbor impacts on the average number of outpatient visits.

**Conclusion:**

Health services utilization was a variable varied by region and should be considered in a geographic context with spatial attributes. From the spatial perspective, this study identified the local and neighbor impacts of predisposing factors, enabling factors, and need factors that contributed to disparities in local health services utilization.

## Introduction

Equity means that there is no unfair, avoidable, or remediable difference between groups, whether in economic, social, demographic, geographical, or otherwise. Health equity can be achieved when everyone has access to health and well-being [1]. Equity in health is an important human right. When considering health-related human rights, the first principle that comes to mind is the "right to health" [2]. To realize this fundamental human right, countries must ensure the availability, acceptability, and quality of healthcare services [3].

However, inequitable access to health services has long been recognized as a problem in many countries [4–6]. In detail, such unfairness is reflected in many aspects, like the economy, geography, demographic characteristics, and health conditions. Access to health services is not only determined by the health status of individuals but is also a consequence of demand generated by socio-economic factors. [7, 8]. Many patients cannot afford the care they need and often forgo medical care altogether for uninsurance or inadequate health insurance [9]. At the same time, access to health services is difficult in many economically disadvantaged areas, and higher levels of medical resources are concentrated in affluent areas. [10, 11]. Furthermore, the utilization of health services has been influenced by age, gender, education, as well as race/ethnicity [8, 12–15]. When the different needs of individuals are not taken into account, inequity might happen [16]. It is believed that people's needs for health care services, and not their various characteristics, must be the basis of access to and utilization of such services [14–17].

To explore the influence of the above factors on health services, scholars have developed various theoretical models for health service utilization, such as stages of illness and medical care, health belief model, and choice-making model [18–20]. However, Andersen's theoretical model is one of the conventionally used models to explain service utilization. Andersen's health service utilization model was developed in western countries and has been extensively tested in various countries [15, 21, 22]. It is a theoretical framework that has been tested by many scholars to explain the determinants of health service use, taking into account individual and societal determinants [21]. The model divides the determinants of health service use into three components: (i) Predisposing factors are primarily the demographic characteristics of the study participants, and these variables may increase or decrease the likelihood that treatment will be considered a viable option or alternative when the patient is faced with the disease; (ii) enabling factors are resources that increase the likelihood that those who need them will be able to seek or use these services; (iii) need factors represents both self-perceived and the actual need for health service [23].

Previous scholars in various countries have used the Andersen’ s Behavioral Model (hereinafter referred to as Anderson Model) to explore the impact of various factors on the utilization of health services [14, 15, 24–27]. According to their research findings, need factors (e.g. health condition and severe degree of symptom, chronic diseases) were deemed to be the predominant determinant of health service utilization. Meanwhile, predisposing factors (e.g. ethnicity, the nature of the working, and age) and enabling factors (e.g. the main source of living, income surplus, and the basic endowment insurance coverage) are also important elements influencing health service utilization. Nowadays, due to the rapid development of transport, a large number of patients seek medical treatment across regions every year, to seek higher-level medical institutions and better medical resources [28–30]. Thus, it could produce spatial spillover effects, which means that observations in one area may be influenced by observations from adjacent areas. Hence, describing the spatial variations of health services utilization and identifying influencing factors of health services utilization would be of great necessity to examine the effects of factors on health services utilization to interpret how factors influence health services utilization disparities. The previous studies on health service utilization usually used ordinary cross-sectional or panel data. However, our research considers spatial attributes, hoping to elaborate on the utilization of health services from a spatial perspective.

Therefore, we used the relevant data from the 2010–2021 China Statistical Yearbook to interpret the utilization of health services in China from a spatial perspective, aiming to (i) describe the spatial distribution of the use of provincial health services in China; (ii) based on Andersen models propose a proxy framework for health services utilization associated predisposing, enabling, and need factors; (iii) quantify the association between factors and health services utilization based on proxy framework; and (iv) analyze the spatial spillover effects of influencing factors in various provinces. The results of this study will help identify influencing factors based on health service utilization at the provincial level and provide evidence for the implementation of region-specific policies to promote health services utilization and improve overall health in China.

## Materials and methods

**Fig. 1:**
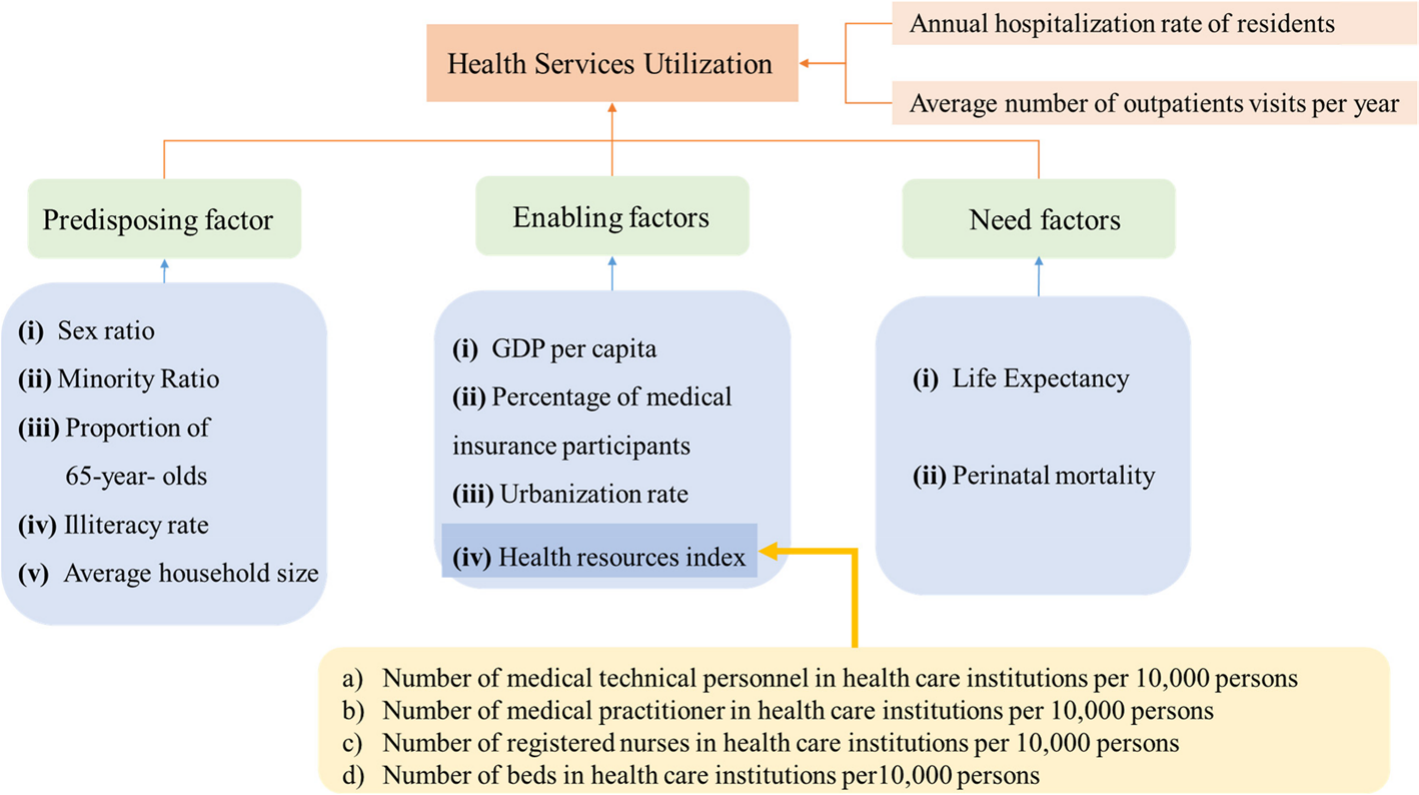
Proxy framework for health services utilization associated predisposing, enabling, and need factors

Our study focused on the factors and their spillover effects on the utilization of province-based health services using the dataset of 31 provinces in China from 2010 to 2020. Data for our study was derived from China Statistical Yearbook 2010–2021. Referring to the influencing factors of the Anderson model, we constructed the proxy framework for health services utilization associated with predisposing, enabling, and need factors (Fig. 1). The analysis software used Arc GIS 10.2 and STATA 16.0.

### Dependent variable

The dependent variables of this study were the province-based health services, including the annual hospitalization rate of residents and the average number of outpatient visits per year.

### Independent variable

Independent variable mainly took into account from the Anderson model, literatures and the available data in statistical yearbooks.

#### Predisposing factors

For predisposition factors, we suggested that age, sex, race/ethnicity, education, and family structure composition may have an impact on the utilization of health service [14, 15, 24–26]. Specifically, it was divided into 5 proxies: (i) Sex ratio (SR, %), which reflects the sex composition. (ii) Ethnic minority groups rate (EMGR, %), which reflects ethnic minority groups composition. (iii) Proportion of 65-year-olds (P65, %), which reflects age composition. (iv) Illiteracy rate (IR, %), which reflects education composition. (v) Average household size (AHS), which reflects the size of the family.

#### Enabling factors

In this component, we included 4 proxies: (i) Per capita gross domestic product (GDP per capita), which reflects the economic contribution or value creation of each resident to their country or region. (ii) Urbanization rate (UR, %), which reflects the proportion of the urban population in the total population. (iii) Percentage of medical insurance participants (MIP, %), which reflects the level of the population enjoying health insurance. (iv) Health resources (HR), which includes a) Number of medical technical personnel in health care institutions per 10,000 persons. b) Number of physicians in health care institutions per 10,000 persons. c) Number of registered nurses in health care institutions per 10,000 persons. d) Number of beds in health care institutions per 10,000 persons. Above four variables, we used principal component analysis (PCA) to convert them into a health resources index.

#### Need factors

We used two indicators: (i) Life expectancy (LE), which is the total number of years that a group of people at birth can expect to live given an existing age-specific mortality rate [31]. (ii) Perinatal mortality (PM), which is the level of perinatal infant mortality.

### Data analysis

#### Descriptive and exploratory spatial data analysis

This paper first described the spatial distribution of the annual hospitalization rate of residents and the average number of outpatient visits per year and calculated the growth rate from 2010 to 2020. We used the local Moran I statistic to examine the spatial autocorrelation between the annual hospitalization rate of residents and the average number of outpatient visits per year. Then, we calculated descriptive statistics (observations, means, standard deviations, min, max, and VIF) to describe predisposing factors, enabling factors, need factors, and utilization of health service variables in 2010–2020.

#### Panel data models

Based on the framework for health services utilization, we first established a general panel regression model to analyze the impact of various influencing factors on the use of provincial health services, the model is represented as follows:

1 $${UHS}_{\mathrm{it}}=\mathrm{c}+{a}_{1}{\mathrm{PF}}_{\mathrm{it}}+{a}_{2}{EF}_{\mathrm{it}}+{a}_{3}{NF}_{\mathrm{it}}+{\mu }_{i}+{\mu }_{t}+{\varphi }_{it}$$

where i denote province, t represents the year, UHS stands for utilization of health service measured by hospitalization rate and the average number of outpatient visits per year. In each region, PF represents the predisposing factor, EF represents the enabling factors, NF represents the need factors, $\mu_i$ denotes the individual (spatial) effect, and $\mu_t$ denotes the time effect respectively, which could be fixed or random effects. $\varphi$ is the error term.

#### Spatial models

Based on Anderson model, we used the spatial panel data model to study the influencing factors of the utilization of provincial health services in China from 2010 to 2020. First, we examined the normality of the annual hospitalization rate of residents and the average number of outpatient visits per year by the Shapiro–Wilk (SW) test. Afterward, based on the panel regression model, we established three spatial regression models: i) the spatial panel autoregressive regression model (SPAR: adding the spatially lagged dependent variable); ii) the spatial panel error model (SPEM: adding the spatially lagged error term); iii) the spatial panel Durbin model (SPDM: adding both spatially lagged dependent variable and spatially lagged error term).

The SPAR takes the form as follows:

2 $${UHS}_{\mathrm{it}}=\mathrm{c}+\rho \sum {W}_{\mathrm{ij}}\times {UHS}_{\mathrm{it}}+{a}_{1}{\mathrm{PF}}_{\mathrm{it}}+{a}_{2}{EF}_{\mathrm{it}}+{a}_{3}{NF}_{\mathrm{it}}+{\mu }_{i}+{\mu }_{t}+{\varphi }_{it}$$

where W denotes the spatial weight matrix, we consider the binary geographic unit matrix = 1 if i and j are neighbors and 0 otherwise. Under the setting of the SPAR model, the health service utilization of a province will be affected by the independent variables of surrounding provinces.

The SPEM fully considers the spatial correlation of error terms. The SPEM is set as follows:

3 $${UHS}_{\mathrm{it}}=\mathrm{c}+{a}_{1}{PF}_{\mathrm{it}}+{a}_{2}{EF}_{\mathrm{it}}+{a}_{3}{NF}_{\mathrm{it}}+{\mu }_{i}+{\mu }_{t}+{\varphi }_{it} {\varphi }_{it}=\sum {W}_{ij}{\varphi }_{it}+{\varepsilon }_{it}$$

$\varepsilon_{it}$ denotes the error term.

SPDM is set as follows:

4 $${UHS}_{\mathrm{it}}=\mathrm{c}+{UHS}_{\mathrm{i}(\mathrm{t}-1)}+\rho \sum {W}_{\mathrm{ij}}\times {UHS}_{\mathrm{it}}+{aX}_{\mathrm{it}}+\sum {W}_{\mathrm{ij}}\times {X}_{\mathrm{it}}\theta +{\mu }_{i}+{\mu }_{t}+{\varphi }_{it}$$

$\theta$ quantifies the spatial spillover effects from spatially lagged independent variables. X denotes predisposing factors, enabling factors, and need factors, $\sum{W_{ij}}\times\text{X}_\text{it}$ representing the spatial lag term of the independent variables.

SPDM considers the influence of both spatial lag-dependent variables and spatial lag-independent variables. Therefore, we performed the Lagrange Multiplier test (LM) to test the rationality of spatial panel model construction of SPAR, SPEM, and SPDM models. and subsequently performed the Wald test, and Likelihood Ratio test (LR) to conduct the model fit evaluation. Then, we first used the LM test (Lagrange Multiplier test), and two dependent variables found that it was appropriate to choose both the SPEM model and the SPAR model, so we initially selected the SPDM model that combines the two (Table 1).

**Table 1:** LM Test result for the utilization of health service

Dependent variable	Test	Statistic	df	*P*-value
Resident hospitalization rate	Spatial error:			
	Moran's I	2.424	1	0.015
	Lagrange multiplier	83.541	1	0.000
	Robust Lagrange multiplier	43.362	1	0.001
	Spatial lag:			
	Lagrange multiplier	62.700	1	0.000
	Robust Lagrange multiplier	22.522	1	0.000
The average number of outpatient visits per year	Spatial error:			
	Moran's I	1.927	1	0.054
	Lagrange multiplier	49.052	1	0.000
	Robust Lagrange multiplier	24.902	1	0.000
	Spatial lag:			
	Lagrange multiplier	36.652	1	0.000
	Robust Lagrange multiplier	12.502	1	0.000

Hausman Test for both the dependent variable of resident hospitalization rate and outpatient visits proved the rationality of using both spatial (individual) and time-fixed effects models. Table 2 concludes that SPDM could not be simplified to SPAR or SPEM for the dependent variable of resident hospitalization rate. Meanwhile, for the dependent variable of the average number of outpatient visits per year, SPDM also could not be simplified to SPAR or SPEM.

**Table 2:** Wald test and LR test result for the utilization of health service

Dependent variable	Model	Test	Statistic	*P*-value
Resident hospitalization rate	SPAR	Wald test	32.26	< 0.001
		LR test	176.33	< 0.001
	SPEM	Wald test	34.41	< 0.001
		LR test	50.81	< 0.001
The average number of outpatient visits per year	SPAR	Wald test	64.88	< 0.001
		LR test	90.35	< 0.001
	SPEM	Wald test	56.15	< 0.001
		LR test	237.63	< 0.001

## Results

### Spatial distribution of utilization of health services in China

**Fig. 2:**
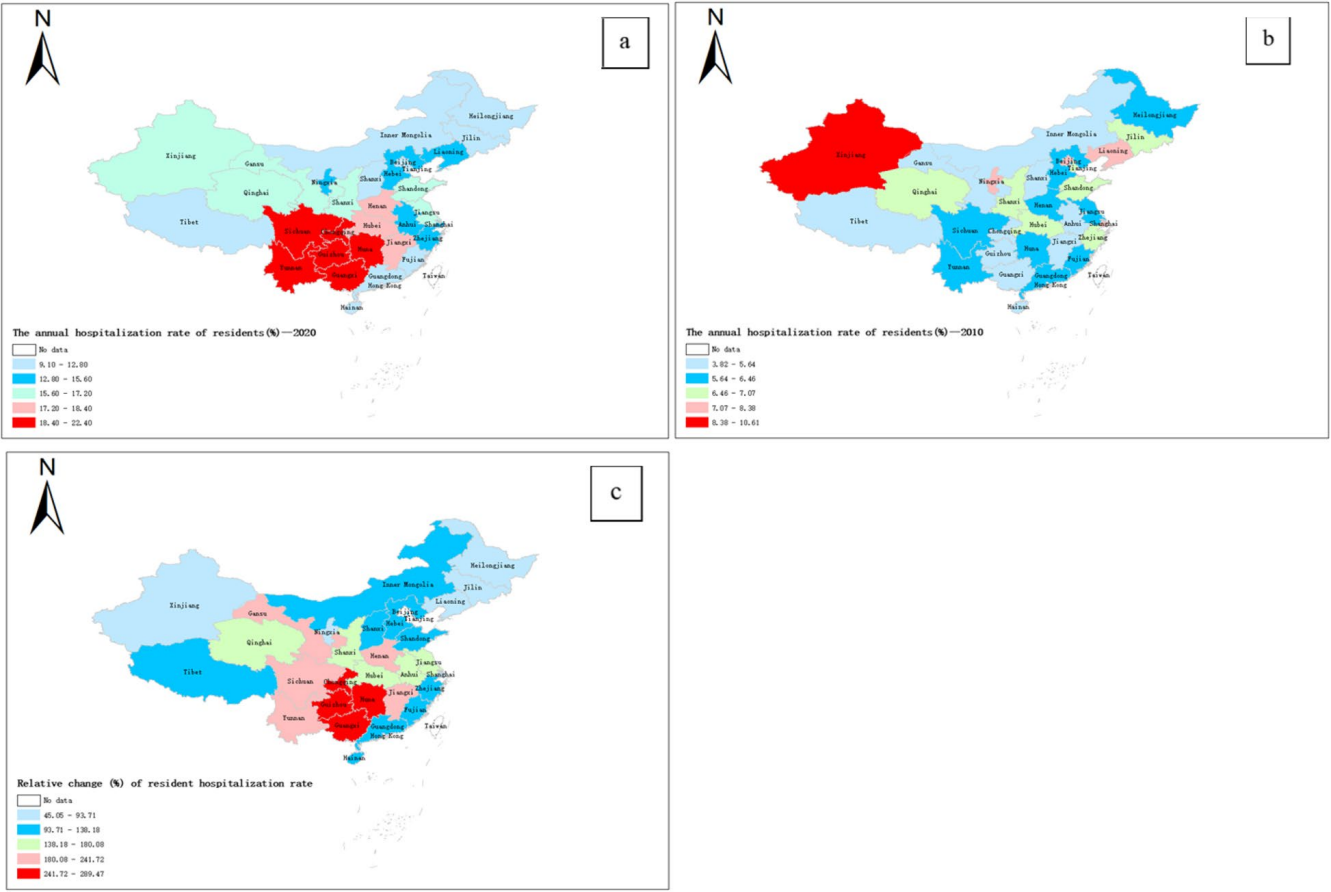
Choropleth map of resident hospitalization rate in China in 2010, 2020 and its relative change during 2010 − 2020. **a**. Resident hospitalization rate in 2010; **b**. The resident hospitalization rate in 2020; **c**. relative change (%) of resident hospitalization rate between 2010 and 2020

During 2010 − 2020, nationwide, the resident hospitalization rate increased from 6.39% ± 1.23% to 15.57% ± 2.61%. Figure 2 shows the top three provinces with the largest increase in hospitalization rates were Chongqing (289.47%), Guizhou (265.74%), and Hunan (264.46%), while the three regions with the lowest growth rates were Tianjin (41.29%), Beijing (45.06%) and Xinjiang (59.41%).

**Fig. 3:**
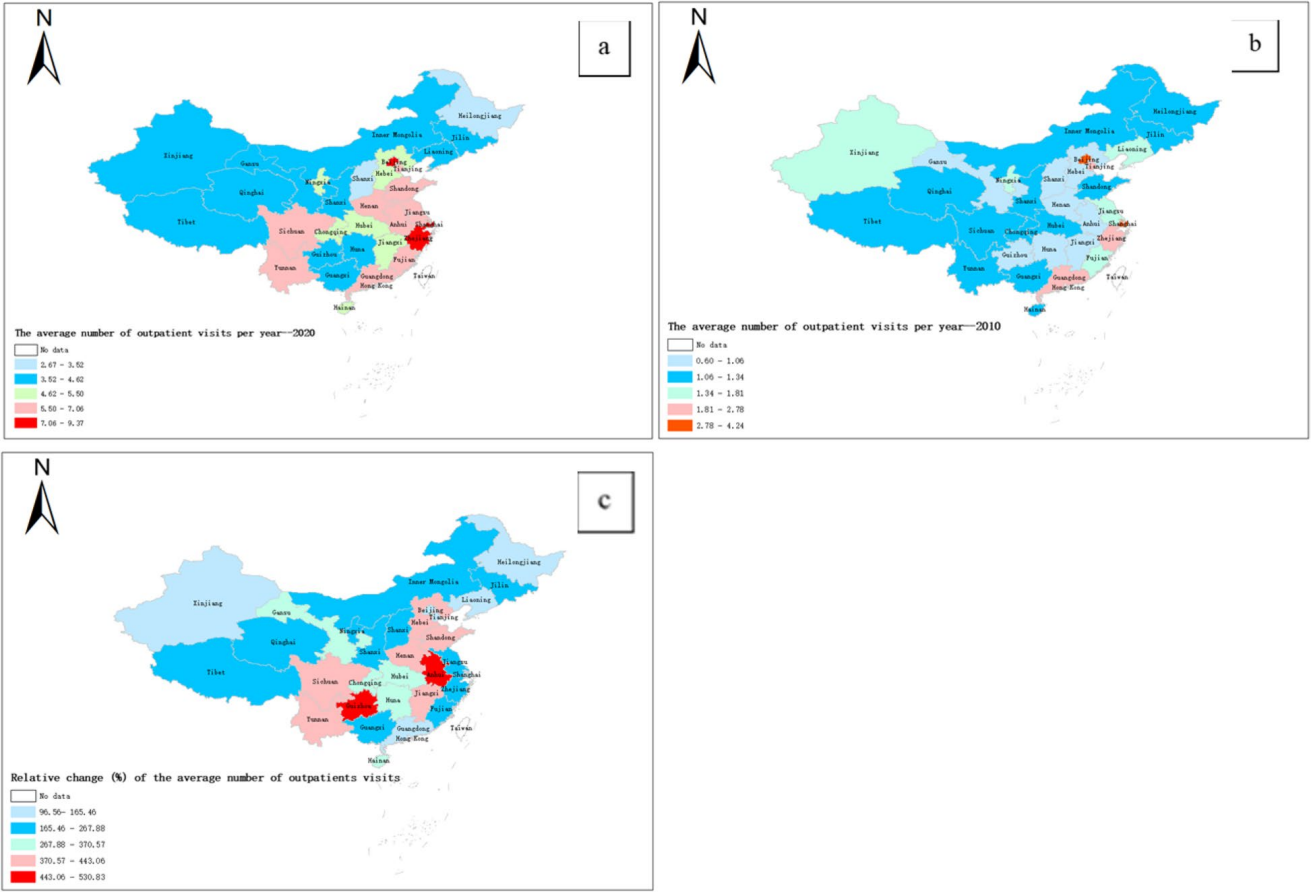
Choropleth map of the average number of outpatients visits per year in China in 2010, and 2020 and its relative change during 2010 − 2020. **a**. The average number of outpatients visits in 2010; **b**. The average number of outpatients visits in 2020; **c**. relative change (%) of the average number of outpatients visits between 2010 and 2020

The average number of outpatient visits per year increased from 1.53 ± 0.86 times to 5.30 ± 1.54 times. Figure 3 shows the top three provinces with the largest increase in the average number of visits were Anhui (530.82%), Guizhou (505.29%), and Henan (443.06%), while the three regions with the lowest growth rates were Beijing (96.56%), Shanghai (116.53%) and Heilongjiang (131.69%).

**Fig. 4:**
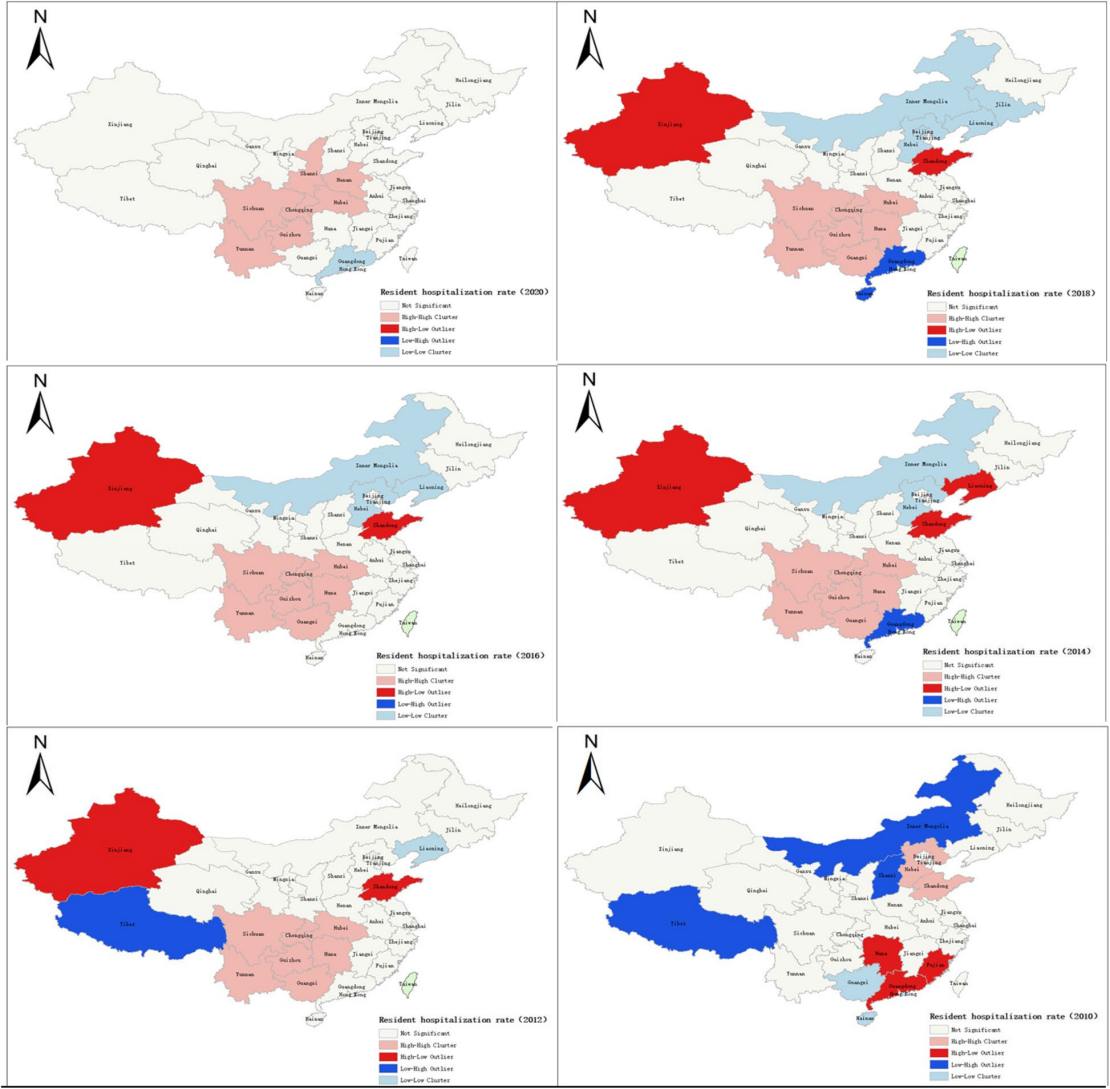
Local Moran I of resident hospitalization rate in China during 2010 − 2020

**Fig. 5:**
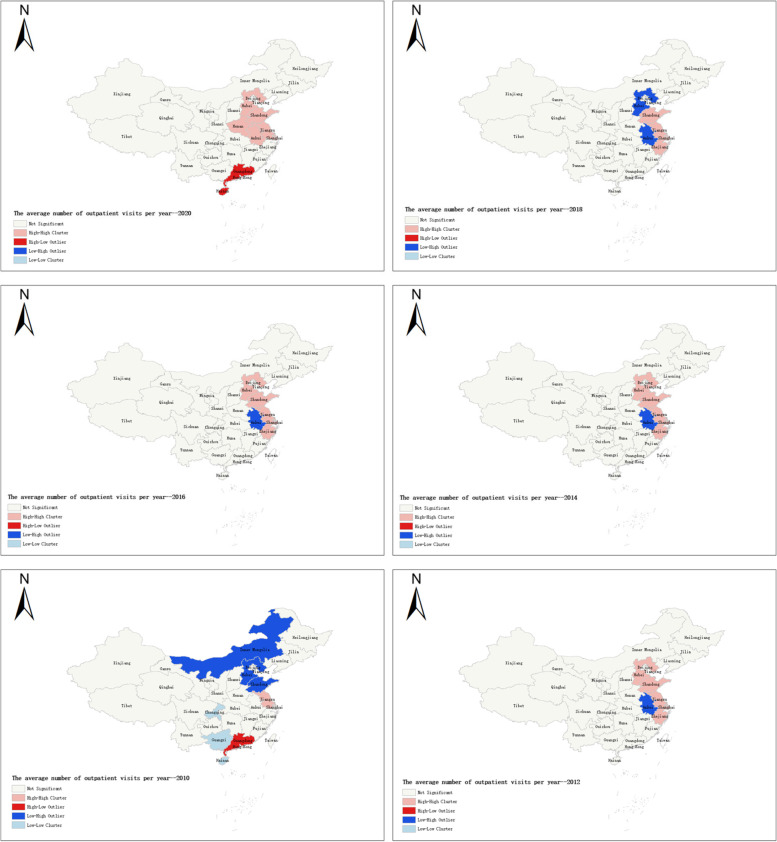
Local Moran I of the average number of outpatient visits per year in China during 2010 − 2020

The spatial autocorrelation test was the first step of spatial econometric analysis. The local Moran I was used to describing spatial uncertainty and detect spatial clustering of the resident hospitalization rate and the average number of outpatient visits during 2010 − 2020.The results of spatial clustering divided 31 provinces into four parts (Figs. 4 and 5):1) High-High (H–H) positive spatial correlation: the provinces with higher utilization of health services were encircled by similar higher provinces, as shown in the pink part of the figure. From 2012 to 2020, Sichuan, Guizhou, Chongqing, Hunan, Hubei, and Guangxi areas are High-High (H–H) positive spatial correlation in resident hospitalization rate; 2) Low–High (L–H) negative spatial correlation: the provinces with lower utilization of health services were encircled by higher provinces, as shown in the red part of the figure.; 3) Low-Low (L-L) positive spatial correlation: the provinces with lower utilization of health services were surrounded by lower provinces, as shown in the blue part of the figure.; 4.) High-Low (H–L) spatial correlation: the provinces with higher utilization of health services were encompassed by lower ones, as shown in the dark blue part of the figure.

### Descriptive analysis using variables

Description of variables for 31 provinces was presented by mean, Std. Err, min value, max value, and VIF from 2010 to 2020 separately. All variables were included in the study because their VIF was less than 10. In the subsequent spatial model, the variables we used are summarized and listed in Table 3. And the Shapiro–Wilk (SW) test result shows that both the annual hospitalization rate of residents and the average number of outpatient visits per year conform to normality.

**Table 3:** Descriptive statistics summarizing predisposing factors, enabling factors, need factors, and utilization of health service variables in 2010–2020

Factors	Variable	Obs	Mean	Std	Min	Max	VIF
Predisposing factor	Sex ratio (SR, %)	186	105.03	3.61	96.91	118.62	1.38
	Ethnic minority groups rate (EMGR, %)	186	32.98	29.39	0	97.45	1.79
	The proportion of 65-year-olds (P65, %)	186	10.46	2.59	4.98	17.41	2.96
	Illiteracy rate (IR, %)	186	5.67	5.90	0.89	41.12	2.44
	Average household size (AHS)	186	2.99	0.41	2.06	4.23	2.45
Enabling factors	Ln (GDP per capita)	186	10.72	0.49	9.27	12.01	3.80
	Percentage of medical insurance participants (MIP, %)	186	59.58	30.65	3.16	99.99	2.36
	Urbanization rate (UR, %)	186	57.48	13.42	22.67	89.30	8.37
	Health resources index (HRI)	186	0.00	1.00	-2.06	4.41	2.80
Need factors	Life Expectancy (LE)	186	76.20	3.10	68.17	83.67	7.51
	Perinatal mortality (PM)	186	6.10	3.33	1.80	24.04	2.55
Health Services Utilization	The annual hospitalization rate of residents(%)	186	13.43	4.44	3.82	22.4	NA
	The average number of outpatient visits per year	186	4.68	2.12	0.69	10.72	NA

### Results of spatial panel analysis

Table 4 presents the estimated results of the SPDM for the dependent variable of resident hospitalization rate. For the predisposing factor, locally, the population in provinces with a higher proportion of 65-year-olds and a lower illiteracy rate was associated with an increase in the resident hospitalization rate. For spatially-lagged effects, namely, a lower proportion of 65-year-olds and a higher illiteracy rate exerted auxo-action on resident hospitalization rate among adjacent proximity. Regarding enabling factors, GDP per capita, percentage of medical insurance participants, and health resources were associated with the resident hospitalization rate increase, but the urbanization rate was associated with the resident hospitalization rate decrease locally. For spatially-lagged effects, a higher GDP per capita and a lower percentage of medical insurance participants had a promoting effect on the hospitalization rate in the surrounding region. And the need factors do not affect the resident hospitalization rate.

**Table 4:** The association between predisposing factors, enabling factors, need factors, and the utilization of health service 2010 − 2020: estimated from spatial panel data models

Variable		Resident hospitalization rate		The average number of outpatients visits	
		PANEL	SPDM	PANEL	SPDM
Predisposing factor	Sex ratio	-0.07(0.07)	-0.01(0.04)	0.02(0.03)	0.03(0.02)
	Minority Ratio	0.06(0.02)^***^	0.02(0.01)	0.01(0.01)	-0.01(0.01)
	Proportion of 65-year-olds	-0.02(0.15)	0.39(0.11)^***^	-0.18(0.06)	0.02(0.04)
	Illiteracy rate	-0.06(0.08)	-0.15(0.07)^*^	0.11(0.03)	0.02(0.03)
	Average household size	2.65(0.84)	0.61(0.70)	0.58(0.35)	0.10(0.27)
Enabling factors	Ln (GDP per capita)	4.68(0.90)	1.54(0.59)^**^	2.55(0.38)	1.06(0.24)^***^
	Percentage of medical insurance participants	-0.01(0.01)	0.03(0.01)^**^	-0.01(0.00)	0.01(0.01)
	Urbanization rate	-0.20(0.06)	-0.15(0.05)^**^	-0.01(0.02)	0.03(0.02)
	Health Resources Index	2.68(0.45)	1.93(0.30)^***^	0.55(0.18)	0.09(0.13)
Need factors	Life Expectancy	0.08(0.23)	-0.05(0.16)	0.25(0.09)	0.07(0.12)
	Perinatal mortality	-0.62(0.09)	-0.15(0.12)	-0.18(0.05)	0.01(0.04)
Constant		-28.44(18.84)	-22.83(29.88)	-43.29(7.70)^***^	-32.29(11.28)^*^
Spatially lagged effects					
Predisposing factor	Sex ratio		0.06(0.09)		0.014(0.04)
	Minority Ratio		0.001(0.03)		0.02(0.01)
	Proportion of 65-year-olds		-0.64(0.16)^***^		-0.24(0.07)^***^
	Illiteracy rate		0.84(0.20)^***^		0.32(0.08)^***^
	Average household size		-0.60(0.98)		-0.83(0.40)
Enabling factors	Ln (GDP per capita)		1.69(0.65)^**^		0.62(0.29)^*^
	Percentage of medical insurance participants		-0.05(0.01)^***^		-0.01(0.00)^*^
	Urbanization rate		0.08(0.09)		-0.05(0.04)
	Health resources index		-0.09(0.47)		0.12(0.19)
Need factors	Life Expectancy		0.01(0.32)		0.14(0.13)
	Perinatal mortality		-0.39(0.21)		-0.12(0.09)
R^2^		0.6170	0.8486	0.5819	0.8688
AIC			792.3303		462.6909
BIC			876.1997		546.5603

*PANEL* Ordinary panel data model, *SPDM* Spatial panel Durbin model with time and space–time lagged utilization of health service in the regressors

^*^ *P* < 0.05 ** *P* < 0.01 *** *P* < 0.001

The results of the average number of outpatient visits by dependent variables are also presented in Table 3. For the predisposing factor, considering spatially lagged effects, the region with a higher illiteracy rate, and a lower proportion of 65-year-olds may promote outpatient visits in adjacent proximity. For the enabling factor, GDP per capita was associated with locally increased outpatient visits. For spatially-lagged effects, a higher GDP per capita and a lower percentage of medical insurance participants had a promoting effect on outpatient visits in the surrounding region. However, life expectancy and perinatal mortality do not affect the average number of outpatient visits.

### Estimation of spatial spillovers

Based on SPDM estimation, we further conducted direct/indirect effects decomposition to interpret how predisposing factors, enabling factors, and need factors to influence the utilization of health service disparities (Table 5).

**Table 5:** The association between predisposing factors, enabling factors, need factors, and the utilization of health service 2010 − 2020: estimated from spatial panel data models

Variable		Direct	Indirect	Total
**Resident hospitalization rate**				
Predisposing factor	Sex ratio	-0.01(0.05)	0.08(0.19)	0.07(0.22)
	Minority Ratio	0.03(0.01)	0.02(0.06)	0.05(0.07)
	Proportion of 65-year-olds	0.34(0.01) ^**^	-0.83(0.27) ^**^	-0.50(0.32)
	Illiteracy rate	-0.04(0.08)	1.39(0.37) ^***^	1.34(0.42) ^**^
	Average household size	0.58(0.68)	-0.43(1.63)	1.50(1.83)
Enabling factors	Ln (GDP per capita)	1.93(0.60) ^**^	4.46(1.19) ^***^	6.39(1.52) ^***^
	Percentage of medical insurance participants	0.02(0.01)^**^	-0.06(0.02)^***^	-0.04(0.02)^*^
	Urbanization rate	-0.16(0.05) ^**^	0.01(0.16)	-0.15(0.18)
	Health resources index	2.09(0.311) ^***^	1.60(0.87)^*^	3.69(1.02) ^***^
Need factors	Life Expectancy	-0.05(0.19)	-0.04(0.71)	-0.09(0.85)
	Perinatal mortality	-0.22(0.13)	-0.89(0.43)^*^	-1.11(0.52)^*^
**The average number of outpatient visits per year**				
Predisposing factor	Sex ratio	0.04 (0.02)	0.06(0.09)	0.09(0.11)
	Minority Ratio	-0.01 (0.01)	0.04(0.03)	0.03(0.03)
	Proportion of 65-year-olds	-0.01(0.04)	-0.49(0.13) ^***^	-0.50(0.15) ^**^
	Illiteracy rate	0.08(0.03)^*^	0.68(0.18)^***^	0.76(0.20)^***^
	Average household size	-0.04(0.27)	-1.57(0.76)^*^	-1.61(0.86)
Enabling factors	Ln (GDP per capita)	1.29(0.25) ^***^	2.50(0.54)^***^	3.79(0.68)^***^
	Percentage of medical insurance participants	0.01(0.01)	-0.02(0.01)	-0.01(0.01)
	Urbanization rate	0.03(0.02)	-0.06(0.08)	-0.04(0.09)
	Health Resources	0.13(0.14)	0.35(0.41)	0.49(0.49)
Need factors	Life Expectancy	0.10(0.08)	0.36(0.31)	0.47(0.37)
	Perinatal mortality	-0.01(0.05)	-0.24(0.20)	-0.25(0.23)

^*^ *P* < 0.05 ** *P* < 0.01 *** *P* < 0.001

#### Spatial spillover effect of predisposing factors

For the proportion of the 65-year-old variable, the direct impact on the hospitalization rate was 0.34, and the indirect impact was -0.83. And for the illiteracy rate, its impact on the hospitalization rate was impacted by indirect effects (1.39) and total effects (1.34). Meanwhile, for the average number of outpatients visits, the impact of the proportion of 65-year-olds on it was also mainly reflected by the indirect effect (-0.49). For the illiteracy rate, the direct, indirect, and total influence values of the number of outpatient visits were 0.08, 0.68, and 0.76 respectively.

#### Spatial spillover effect of enabling factors

The direct, indirect, and total effects of the logarithm of GDP per capita on the hospitalization rate were 1.93, 4.46, and 6.39, and the number of outpatient visits were 1.29, 2.50, and 3.79. Meanwhile, for every 1% increase in the percentage of medical insurance participants, the direct effect of local hospitalization will increase by 0.02%, the adjacent area will decrease by 0.06%, and the total effect will decrease by 0.04%. For the health resources index rate, the direct, indirect, and total influence values of the resident hospitalization rate were 2.09, 1.60, and 3.69 respectively.

#### Spatial spillover effect of need factors

For perinatal mortality, its impact on the hospitalization rate was impacted by indirect effects (-0.89), and total effects( -1.11).

## Discussion

By using data from China Statistical Yearbook (2010–2021), this study provided comprehensive estimates of health services utilization at the provincial level in China. We found health services utilization was a variable varied by region and should be considered in a geographic context.

### Spatial variations of health services utilization

During 2010 − 2020, the overall health services utilization in China has risen steadily, which was closely related to improvement in socioeconomic and medical conditions, new policy, health care reform and population aging, and social health insurance system [32–34]. The increase in the use of health services in the southwest region, especially the hospitalization rate, is more pronounced and clustered. This may be mainly because since 2010, the population gravity centers and economic gravity centers have moved to the southwest [35], and economic development and population movement have promoted the use of health services. At the same time, the utilization of health services in northern is generally lower than that in southern China, and this difference may be due to the medical efficiency gradual enhancement from north to south [36].

### Health services utilization associated predisposing factors

When other conditions remain unchanged, for every 1% increase in the proportion of 65-year-olds, the local hospitalization rate will increase by 0.34%, while the hospitalization rate in nearby areas will decrease by 0.83%. Older adults have more medical needs [37, 38]. Nevertheless, due to the limitations of the physical condition for the elderly, they are more inclined to choose hospitals in their province. And illiteracy rate indirectly has a positive impact on the hospitalization rate. In other words, the local illiteracy rate increased by 1%, bringing a 1.39% growth in the adjacent hospitalization rate. The possible reason is that provinces with much lower education people often have relatively backward economic development and medical level, so local residents are more likely to seek medical treatment in surrounding provinces [39].

For the utilization of outpatient services, the local proportion of 65-year-olds increases by 1%, reducing the outpatient visits in adjacent areas by 0.49. The main reason is similar to the explanation of the hospitalization rate. And, the illiteracy rate has a promoting effect (direct, indirect, and total effect) on the average number of outpatient visits, possibly because local economic development and medical level.

### Health services utilization associated enabling factors

Our study also shed light on the domain of enabling factors that had the largest direct and indirect impacts on provincial-level health service utilization. Under other conditions, if the logarithm of GDP per capita increases, the local, adjacent areas, and the total health services utilization (both hospitalization rate and the number of outpatient visits) will increase. The impact of per capita GDP on hospitalization and the number of outpatient visits were consistent with previous studies [40–42]. The local economic increase might promote the development of the surrounding region and thus generate positive impacts on GDP, then subsequently increase health services utilization in related regions. The percentage of medical insurance participants has a promoting effect on the local area, but an inhibiting effect on the adjacent areas and the total impact. Higher insurance participation means that more residents are reimbursed for medical treatment, which in turn allows residents to spend less on medical treatment and better access to health services. But the impact of medical insurance participants on neighboring provinces may be due to high health expenses and the inconvenience of inter-provincial medical insurance reimbursement in the past few decades, so residents are more inclined to use medical insurance services in their province [14]. The results of the health resource index show that health resources can promote the hospitalization rate in local and adjacent areas. In other words, provinces with rich health resources can not only provide adequate inpatient services for their residents but also provide inpatient services for neighboring provinces. The possible reason is that when residents need hospitalization services, the disease severity is often high, so they prefer to choose hospitals with more abundant medical resources.

### Health services utilization associated Need factors

In our study, we found that the local perinatal mortality rate will increase by 1 unit, which can inhibit the hospitalization rate in adjacent areas. The reason for the result may be that the perinatal mortality rate is largely affected by socioeconomic status [43–46]. A higher perinatal mortality rate in this region means a poorer economic situation, which will also have a negative impact on the surrounding economy, may bring to a higher perinatal mortality rate in the surrounding areas. The above phenomenon also reflects their weak awareness of neonatal care and lack of awareness of seeking better medical resources in other provinces. However, more specific reasons need further study.

The influencing factors of the outpatient visits per year varied not change with the influencing factors of the resident hospitalization rate, which also validated that the association between the number of outpatient visits and the number of inpatient episodes was not the same [47].

## Strengths and limitations

According to our knowledge, this paper is the first to study the utilization of provincial health services and its influencing factors from a spatial perspective at provincial level. By referring to the influencing factors of Andersen Model, we established the framework for health services utilization. Our study may provide evidence for the implementation of region-specific policies to promote health services utilization and improve overall health in China.

The current study has three limitations. First of all, there may exist an ecological fallacy in this study, because this study can only explain how the influencing factors of health service utilization operate at the provincial level, and cannot establish robust evidence of causality. Second, the variables selected based on the predisposing factor, enabling factors, and need factors in Andersen model may be incomplete, hence omitted variables and potential confounders were inevitable. Third, due to the limitation of data acquisition, this study selected perinatal mortality as the demand factor, which has the defect of index selection.

## Conclusion

Our research found that there are differences in the utilization of health services at the provincial level in China, and the difference is mainly impacted by the predisposing factor (proportion of 65-year-olds and illiteracy rate) and enabling factors (GDP per capita, medical insurance participants, and health resources).

Improving equality of health service utilization is the basis for realizing health equity [48]. Moreover, justice and equity are one of critical principles in Healthy China 2030 [49]. The utilization of health services may interplay between various factors. Thus, based on the results of this article, we propose the following suggestions: 1) There are differences in the utilization of health services among areas, which should be formulated regional-specific strategies to narrow the gap; 2) Policies on the use of health services should be skewed towards the region with large proportion of low-educated and older population; 3) The state has adjusted the reimbursement policy in different provinces, so it is more convenient to improve the hospitalization rate in adjacent areas. 4) Vigorously develop the regional economy, improve the economic level of provinces.

## Data Availability

Publicly available data were analyzed in this study. This data can be found here: STATISTICAL YEARBOOK OF CHINA. (http://www.stats.gov.cn/tjsj./ndsj/).
